# Correction: Albendín-García et al. Explanatory Models of Burnout Diagnosis Based on Personality Factors in Primary Care Nurses. *Int. J. Environ. Res. Public Health* 2022, *19*, 9170

**DOI:** 10.3390/ijerph20156479

**Published:** 2023-07-31

**Authors:** Luis Albendín-García, Nora Suleiman-Martos, Elena Ortega-Campos, Raimundo Aguayo-Estremera, José L. Romero-Béjar, Guillermo A. Cañadas-De la Fuente

**Affiliations:** 1 Casería de Montijo Health Center, Granada-Metropolitan Health District, Andalusian Health Service, Calle Virgen de la Consolación, 12, 18015 Granada, Spain; lualbgar1979@ugr.es; 2 Faculty of Health Sciences, University of Granada, Avenida de la Ilustración, 60, 18016 Granada, Spain; norasm@ugr.es (N.S.-M.); gacf@ugr.es (G.A.C.-D.l.F.); 3 Department of Psychology, University of Almería, 04120 Almería, Spain; elenaortega@ual.es; 4 Department of Psychobiology and Methodology in Behavioral Sciences, Complutense University of Madrid, Campus de Somosaguas, 28223 Pozuelo de Alarcón, Spain; raaguayo@ucm.es; 5 Department of Statistics and Operations Research, University of Granada, Fuentenueva s/n, 18071 Granada, Spain; 6 Instituto de Investigación Biosanitaria (ibs. GRANADA), 18012 Granada, Spain; 7 Institute of Mathematics of the University of Granada (IMAG), Ventanilla 11, 18001 Granada, Spain; 8 Brain, Mind and Behaviour Research Center (CIMCYC), University of Granada, 18071 Granada, Spain

The following corrections have been made to the paper [1]:

## Text Correction

(1) The acronym PA is first defined as “*low personal accomplishment*” (Section 1, paragraph 2); however, it is later used to mean “*personal accomplishment*” in the rest of the paper. All occurrences in the article of acronyms have been standardized according to their first definitions to avoid this problem.
(2) In the last paragraph of Section 3.3 and the first of Section 4, terms expressing an increase (such as high, greater, and increasing) have been modified when the proper meaning was a decrease (such as low, lower, and decreasing).

The corrected paragraph in Section 3.3 is as follows:

Odds ratios (see Tables 3–5) for the significant variables can be interpreted as measures of strength related to increasing or decreasing severity in each dimension. All of the considered personality-related variables are discrete variables; therefore, it is not expected for relevant changes to occur with just one unit of increase in these variables (the odds ratios are close to one), but with more units of increase. In fact, if Fr increased by six units, the odds ratio referred to as moving to a low level of EE (to a low level of DP and PA) would be 1.45 (1.63 and 1.47, respectively) times greater than if Fr did not increase. The opposite effect took place with Nt in EE and DP. Thus, if Nt decreased by six units, the odds ratio of passing to a low level of EE (DP) would be 2.09 (1.4). Finally, an increase of six units in the value of Ry produced an odds ratio of moving to a low level of 1.83 times greater for PA and 1.33 times greater for DP, whilst an increase of six units in the value of Ex involved odds ratios of passing to low levels of 1.39 greater for both PA and EE. It is important to highlight that none of the confidence intervals of the odds ratios for changes of six units in the significant variables contained a value of 1.

The corrected paragraph in Section 4 is as follows:

The objectives of this study were to identify risk factors related to personality variables and quantify their effects for prognosis at the different levels of each dimension of burnout syndrome for PC nurses. With regard to the first objective and the personality-related variables, three models that provide a first approximation of level changes in each of the three dimensions of burnout syndrome were obtained. These models included all of the variables related to personality as explanatory variables. Friendliness is a protective factor included in the three models, and consequently it is involved in level changes for the three dimensions of burnout syndrome. Ry is included in the models related to depersonalization and low personal accomplishment; therefore, it is a relevant variable involved in level changes for these two dimensions. Ex is involved in level changes associated with the dimensions related to EE and PA, whilst Nt is involved in the models related to EE and DP being relevant in their level changes. These models are able to predict the probability of an individual being at a burnout level, according to changes in the explanatory variables. With regard to the second objective, the results showed that high values of Fr were associated with situations of lower burnout severity in the three dimensions of this syndrome. In the same way, Ry is a protective factor involving decreasing burnout severity in the DP and PA dimensions, and Ex is also a protection factor in the same way in the EE and PA dimensions. In contrast, higher values of Nt were associated with increasing burnout severity in the EE and DP dimensions.

(3) The sentence “Nt and Op are included in the models related to emotional exhaustion and depersonalization” in the first paragraph of Section 4 has been replaced by “Ry is included in the models related to depersonalization and low personal accomplishment”, which represents the information given in Section 3.

## Error in Figures

The placements of Figures 4 and 5, which were exchanged, have been updated.

## Error in References

The placements of references 9 and 10, which were exchanged, have been updated.

## Error in Authorship

One of the researchers, José A. Sáez, was mistakenly included among the authors of the article; he did not meet the authorship criteria, and his name has therefore been removed. The corrected Author Contributions statement appears here.

**Author Contributions:** Conceptualization and Investigation, G.A.C.-D.l.F. and L.A.-G.; Formal analysis, J.L.R.-B. and E.O.-C.; Methodology, E.O.-C. and R.A.-E.; Resources, N.S.-M.; Software, J.L.R.-B.; Supervision, G.A.C.-D.l.F.; Validation, R.A.-E.; Visualization, E.O.-C., G.A.C.-D.l.F., L.A.-G. and R.A.-E.; Writing—Original draft, L.A.-G. and N.S.-M.; Writing—Review and Editing, J.L.R.-B. and G.A.C.-D.l.F. All authors have read and agreed to the published version of the manuscript.

The authors state that the scientific conclusions are unaffected. This correction was approved by the Academic Editor. The original publication has also been updated.
